# Symmetric accelerating beam generation *via* all-dielectric metasurfaces

**DOI:** 10.1039/d0ra04584e

**Published:** 2020-08-17

**Authors:** Hammad Ahmed, Arbab Abdur Rahim, Muhammad Mahmood Ali, Husnul Maab

**Affiliations:** Ghulam Ishaq Khan Institute of Engineering Sciences and Technology Swabi 23460 Pakistan arbab@giki.edu.pk hammmaad.ahmed@gmail.com; Optical Fibre Sensors Research Centre, Department of Electronics and Computer Engineering, University of Limerick Limerick V94 T9PX Ireland

## Abstract

Traditionally, symmetric accelerating beam (SAB) generation requires bulky optical components, which hinder the miniaturization of optical systems. Recently, metasurfaces, which are composed of sub-wavelength features, have provided a captivating boulevard for the realization of ultra-thin and flat optical devices. Therefore, for the first time, we design and simulate all-dielectric metasurfaces based on an optical caustic approach to generate highly efficient SABs by tailoring the phase of an incident wave. The designed metasurface utilizes spatial distribution of optimized Nb_2_O_5_ nano-rods on SiO_2_ substrate to perform the phase modulation. In contrast with conventional accelerating beams, the generated SABs can follow any predefined propagation trajectory with unique features, such as symmetric intensity profile, autofocusing, and thin needle-like structure in their intensity profile. In addition to this, these beams have also shown the ability to avoid obstacles, placed in the direction of propagation of main lobes. We believe that these beams can be useful in applications, including Raman spectroscopy and fluorescent imaging, and multiparticle manipulation.

## Abstract 2

In this article, all-dielectric metasurfaces are proposed to generate symmetric accelerating beams by manipulating the phase of the incident light.

## Graphical abstract

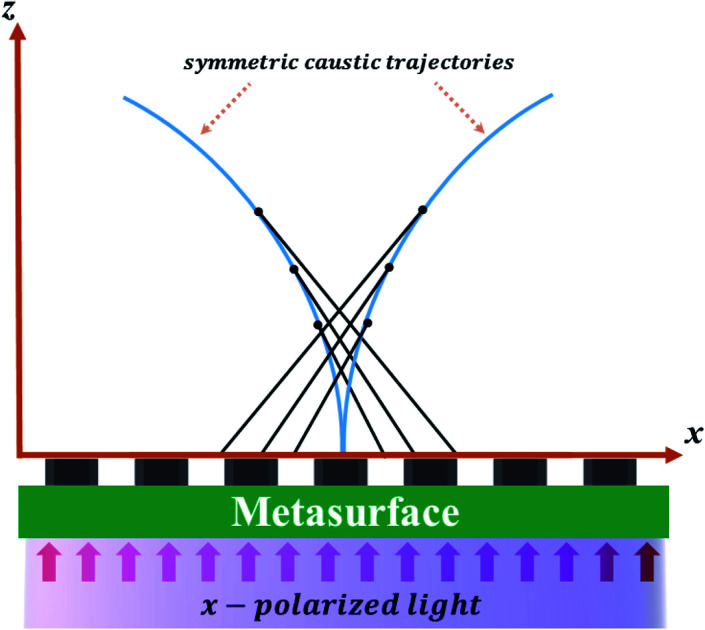

## Introduction

Over the past few years, accelerating beams have attracted the attention of researchers owing to their unprecedented properties, *i.e.*, non-diffraction, self-acceleration, and self-healing. Inspired by Berry and Balazs' work,^1^ these beams were first experimentally demonstrated in 2007.^2^ Since then, self-accelerating beams are considered as an important area of research in the field of optical beams and particle manipulation. The conventional method to generate these beams includes a spatial light modulator (SLM) to impart the desired phase mask on an incident beam.^3^ However, this method usually required a Fourier Transform (FT) lens which makes the optical system bulky and thus, difficult to incorporate in photonic integrated circuits (PICs).^4^ Moreover, the larger pixel size of SLM affects the performance of optical systems.^5^

Metasurfaces, two-dimensional metamaterials provide a remarkable platform to replace the conventional accelerating beam generators. Metasurfaces have intriguing capabilities to mould the incident light to any desired form. By the virtue of subwavelength nano-resonators (unit cells), metasurfaces can tailor the polarization, phase, and amplitude of impinging light to generate high-quality accelerating beam and overcome the limitations imposed by the SLM. Furthermore, several exotic phenomena have been realized through metasurfaces, such as hologram,^6,7^ meta-lens,^8,9^ optical vortex,^10^ meta-polarizer,^11–13^ sensing,^14^ and beam splitter.^11,15^ Recently, numerous metasurfaces have been proposed to demonstrate the accelerating beam generation. Li *et al.*,^4^ Song *et al.*,^16^ Xu *et al.*,^17^ and Wang *et al.*^18^ have utilized the approach of simultaneous phase and amplitude modulation to generate the accelerating beams *via* plasmonic transmission-type metasurfaces. Subsequently, in another approach, Wang *et al.*,^19^ has employed the cubic phase on dielectric metasurfaces and generated the accelerating beam at the back focal plane. However, it is worth mentioning here that most of these beams typically follow a fixed parabolic trajectory, which limits its robustness for practical applications. Therefore, to outclass this drawback, Ahmed *et al.*,^20^ Guo *et al.*,^21^ and Fan *et al.*^5^ have utilized the optical caustic approach to realize the accelerating beams, which can propagate along arbitrary directions. Since then, the control of propagation trajectory has become increasingly interesting area of research.

Inspired from the previous work, herein, we propose single-layered, ultra-thin, all-dielectric metasurfaces for symmetric accelerating beam (SAB) generation. This idea was first theoretically presented in ref. 22. Later on, in 2014 and 2019, the SABs were realized for the first time by utilizing SLM.^23,24^ As discussed earlier, to overcome limitations of SLM, metasurfaces are the best choice. Here, phase profiles of SABs are derived using the optical caustic approach. Then, the derived phase profiles are spatially distributed on metasurface through niobium pentoxide (Nb_2_O_5_) nano-rods. The Nb_2_O_5_ is chosen because it has low absorption at 355 nm (UV domain) compared to other state-of-the-art materials such as titanium oxide (TiO_2_), silicon nitride (Si_3_N_4_), amorphous silicon hydrogenated (a-Si:H) and gallium nitride (GaN).^25,26^ This unique property of low absorption is responsible for high transmission efficiency.^27^ In this work, the phase manipulation of an incident linearly polarized light is performed by varying radii of well-optimized nano-rods while maintaining the high transmission efficiency. Nano-rod geometry is adopted to achieve the polarization-insensitive feature.^28^ This approach is effective in a way that it can alleviate challenges of conventional SLM base SAB generator and can straightforwardly be downsized for on-chip applications. Moreover, symmetric intensity pattern of SAB can manipulate the multiple particles. This property can circumvent requirement of alignment system between the beam and particles.^22^ Thus, simplifying experimental setup for trapping the micro-particles. Besides, SABs also contain a needle-like structure in its intensity profile, which can be used in fluorescent imaging and Raman spectroscopy.^29,30^ Furthermore, we also show the autofocusing characteristic of SABs, which can be used in laser ablation and nano-surgery.^24^

## Design methodology

**Fig. 1:**
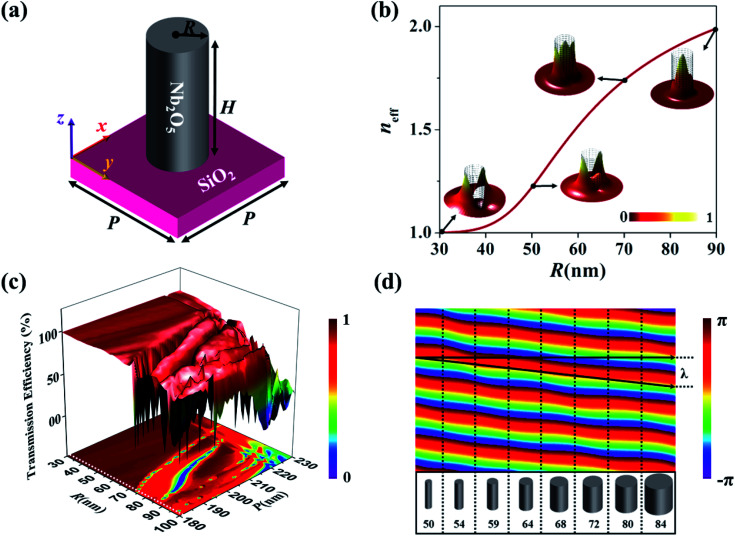
Unit cell design. (a) Schematic depiction of Nb_2_O_5_ nano-rod on SiO_2_ substrate. (b) *n*_eff_ of propagation mode as a function of radius. Insets show the confinement of propagation mode inside the nano-cylinder at some selected radii (*R* = 30, 50, 70 and 90 nm). The cylindrical grid indicates the boundary of a Nb_2_O_5_ nano-rod. (c) Transmission efficiency map. The white dashed line sketched on the map (at *P* = 182 nm with *R* ranging from 30 nm to 100 nm) where the average transmission efficiency is greater than 85%. (d) Complete 2π phase coverage and control over the wave-front is achieved by an array of eight nano-rods of different radii with the fixed height of 430 nm.

To design all-dielectric metasurfaces, nano-resonators with varying geometric parameter are spatially distributed at the interface to achieve full control over an incident wave. In this regard, we chose the nano-rod of Nb_2_O_5_ with a fixed height on SiO_2_ (glass) substrate whose schematic diagram is illustrated in Fig. 1(a). Here, *R* is the radius of nano-rod, *P* represents the periodicity, and *H* is the height. The realization of any phase-sensitive optical phenomenon *via* metasurfaces necessitates that each nano-rod should impart a defined phase to substantiate the desire optical response. Therefore, we optimize our nano-rod in such a way to attain the complete 2π phase coverage while maintaining the maximum possible transmission efficiency (85%). Here, the nano-rod act as a truncated waveguide which works on the wave-guiding effect.^31^ This effect can be better explained through the following relation:^32^1
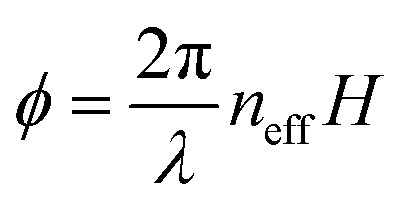
where *λ* is the operating wavelength, *n*_eff_ is an effective refractive index of propagating mode, and *H* is the height of nano-rod. In this, the complete phase coverage is attained by altering the radius of nano-rod. By varying the radius of nano-rod, the effective refractive index (*n*_eff_) of the propagation mode is altered. Fig. 1(b) shows the calculated *n*_eff_ of a propagation mode as function of radius. From the insets, it can be observed that, as the radius of nano-rod increases, the confinement of propagation mode increases, which result in the fine control over the phase of an incident wave.^33^

To confirm complete phase modulation, the numerical optimization of the unit cell is performed through the Finite-Difference-Time-Domain (FDTD) solver. The unit cell is simulated with Perfectly-Matched-Layered (PML) boundary conditions in *z*-axis while periodic boundaries in *x*- and *y*-axis. The *x*-polarized light is impinged from the backside of the unit cell. The optimization results reveal that *P* of 182 nm, *H* of 430 nm and range of *R* from 30 nm to 90 nm can acquire the complete (0–2π) phase coverage (as shown in Fig. 1(d) along with high transmission efficiency (Fig. 1(c))).

**Fig. 2:**
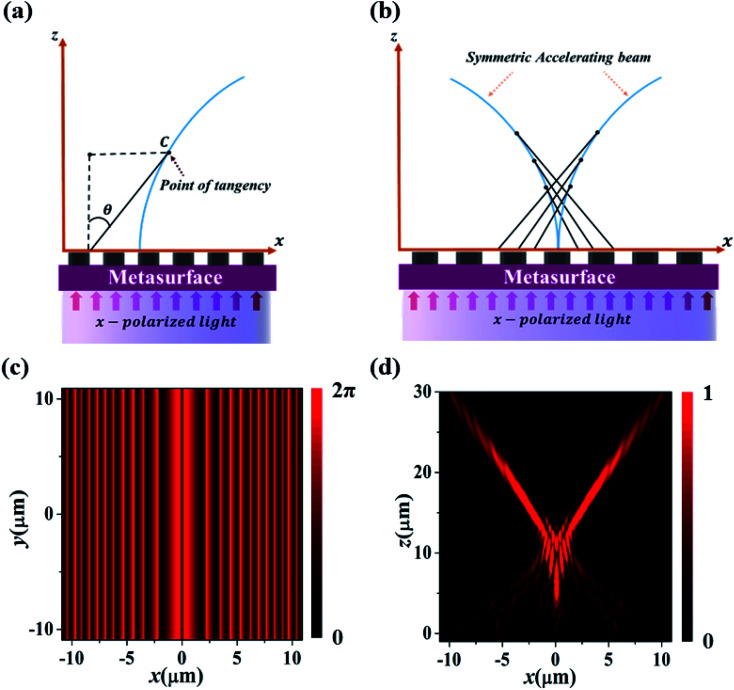
Proposed model based on an optical caustic approach. (a) Asymmetric self-accelerating beam generation. (b) Symmetric self-accelerating beam generation. (c and d) SAB generation with parabolic propagation. (c) Calculated phase profile. (d) Simulated electric field distribution in *x*–*z* plane.

Fig. 2 shows the working principle of our accelerating beam generating metasurfaces where nano-rods of 430 nm height are spatially distributed on a SiO_2_ substrate. The metasurfaces is illuminated with an *x*-polarized plane wave to generate the desired self-accelerating beam. The main aim is to encode a metasurface with a suitable phase profile *ϕ*(*x*), that can generate a self-accelerating beam with predefined propagation trajectory *x* = *f*(*z*). Fig. 2(a) elucidates the schematic diagram of our model. It demonstrates that an accelerating beam can be treated as an optical caustic, that can be described by the family of rays (tangent to the caustic surface) emanating from the metasurfaces, which eventually converges to a focal line *x* = *f*(*z*). Each point on metasurfaces can be functionally related to the corresponding point on optical caustic through the tangent of a slope. Therefore, the slope of optical caustic at an arbitrary point *C* can be defined as 
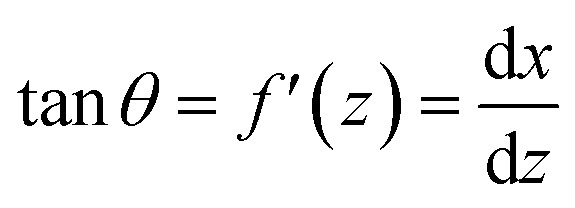
. Whereas, the desired phase that can be spatially distributed along metasurfaces is given as:^34^2
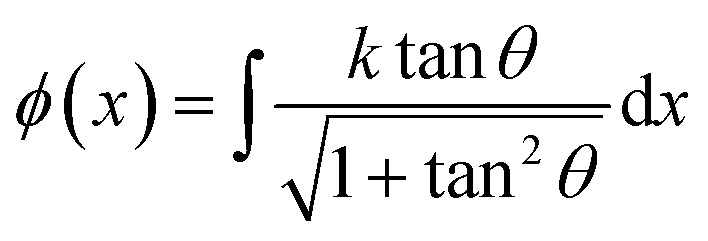
Here, 
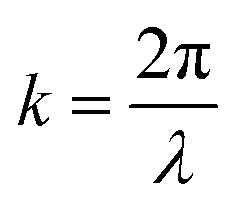
 is the wavenumber and *λ* is the operating wavelength. Eqn (2) can be further simplified by assuming paraxial approximation, because, in current form, it is difficult to solve this integration analytically. Consequently, the simplified case can be given as:3
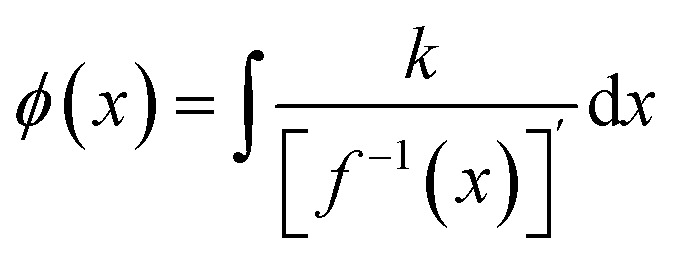
here, *f*^−1^(*x*) is inverse of *f*(*z*).

To design a metasurface capable of generating SAB, herein, the mirror-symmetry of phase *ϕ*(*x*) is performed, *i.e.*, *ϕ*_SAB_(*x*) = *ϕ*(|*x*|). The *ϕ*_SAB_(*x*) corresponds to the symmetric phase profile. The phase profile *ϕ*_SAB_(*x*) shows a perfect symmetry about the optical axis because *ϕ*_SAB_(*x*) = *ϕ*_SAB_(−*x*) is an even function.^24^ Therefore, when metasurface is encoded with *ϕ*_SAB_(*x*), a SAB will be generated as shown in conceptual Fig. 2(b). In contrast to conventional accelerating beam, SAB possesses a symmetric intensity profile which is composed of two separate accelerating beams, that can manipulate multiple particle. Therefore, such highly structured beams can simplify the experimental setup for optical tweezers, as the symmetric intensity pattern overcomes the requirement of alignment system between the beam and particle.^22,24,35^

## Numerical results and discussion

**Fig. 3:**
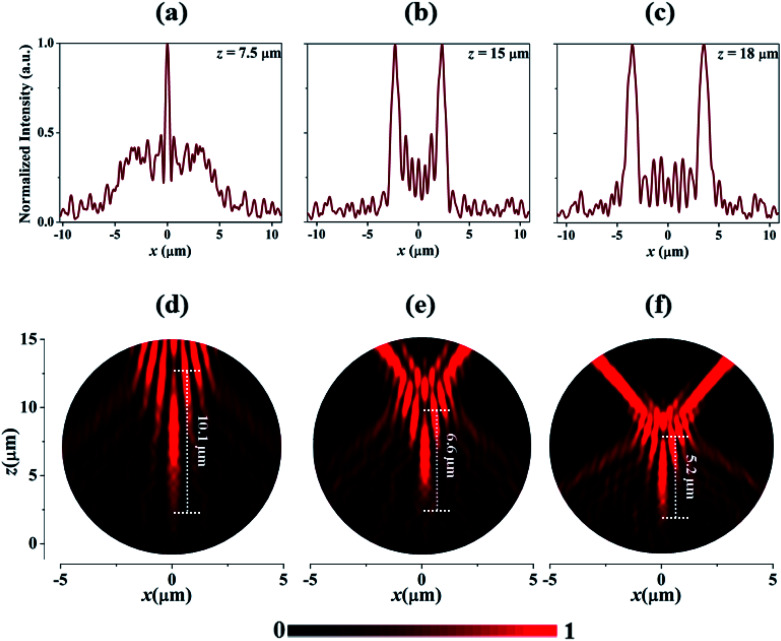
Normalized intensity profiles recorded at (a) *z* = 7.5 μm, (b) 15 μm, and (c) 18 μm. Simulated electric field distributions of thin needle like structure SABs for (d) *a* = 0.012 (e) *a* = 0.009 and (f) *a* = 0.006.

First, we consider the SABs propagating along the parabolic path (*x* = ±*az*^2^). Then, from eqn (3), the corresponding phase distribution for parabolic propagation is calculated, which can be expressed as:4
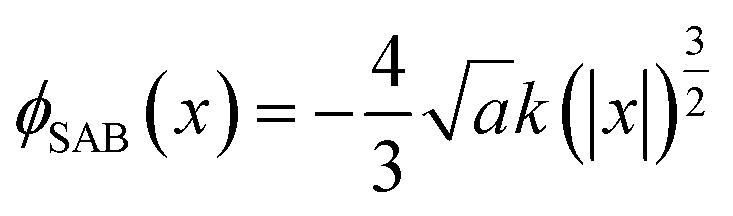
where *a* = 0.009 is the coefficient of acceleration, *k* is the wavenumber, while *x* is the transverse coordinate demonstrating the location of nano-rods. Eqn (4) provides the specific phase profile which is displayed in Fig. 2(c). This phase profile is discretized and implemented onto the 22 μm × 22 μm metasurface by adjusting the radii of nano-rods. Then, the numerical simulation of the metasurface is performed in FDTD solver. The metasurface is subjected to PML boundary conditions in *x*, *y*, and *z* directions. An *x*-polarized plane wave is impinged normally from the substrate side and electric fields are recorded in the simulation domain in the *x*–*z* plane. Fig. 2(b) depicts the simulated electric field intensity distribution of SAB with a parabolic trajectory. It is worth mentioning here that, during the initial stages of propagation, most of the electric field intensity is concentrated inside central lobe. Thereafter, the central lobe collapses, and then, splits into two off-axis self-accelerating beams propagating in the opposite direction. This can be further elaborated through normalized intensity plots which are shown in Fig. 3(a–c). These profiles are plotted by placing field monitor at *z* = 7.5 μm, 15 μm, and 18 μm, respectively. It can be seen that, at *z* = 7.5 μm, the central lobe has the maximum intensity which, later split into two symmetric accelerating beams, propagating in opposite directions.

Additionally, a needle-like structure can be observed in electric field intensity distribution, which is formed by the intensity profile of central lobe. The coefficient of acceleration ‘*a*’ is the key parameter in eqn (4) for controlling the length of the needle. Fig. 3(d–f) illustrate the thin needle-like structure of SAB for *a* = 0.006, 0.009, and 0.012, respectively. The larger value of ‘*a*’ produce a shorter needle and *vice versa*. This fascinating property can help in several important applications, such as fluorescent imaging and second-harmonic generation.^30^

Furthermore, we extend this approach to generate autofocusing SAB. To realize this, we have to perform the phase shift. The modified phase equation with phase shift factor can be expressed as:

5 *ϕ*_SAB_(*x*) = *ϕ*(|*x*| + Δ*x*)

**Fig. 4:**
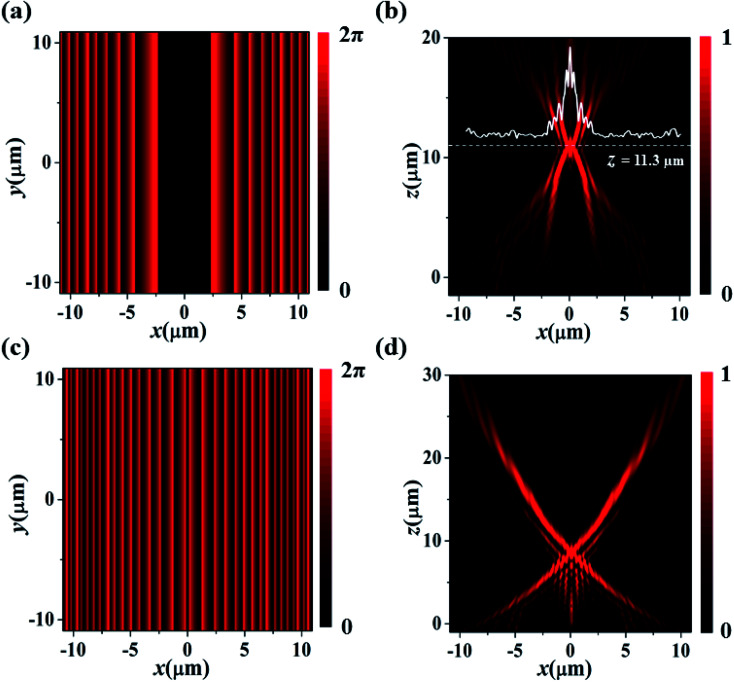
Autofocusing SAB. (a) Shifted phase profile. (b) Simulated electric field intensity distribution in *x*–*z* plane. The solid white line indicates the normalized intensity profile at focal point (*z* = 11.3 μm). (c and d) SAB generation with natural logarithmic trajectory. (c) Calculated phase profile. (d) Simulated electric field distribution in *x*–*z* plane.

Here, Δ*x* is the shift factor in the *x*-direction. Fig. 4(a) illustrates the shifted phase profile of parabolic SAB. The metasurface design methodology is the same as discussed earlier. Fig. 4(b) depicts the simulated electric field intensity. The accelerating beam auto focus at a distance *z* = 11.3 μm, without utilizing an additional lens. This approach of focusing light beam can be employed in optical trapping and light-bullets generation.

As mentioned previously, SABs are optical caustics, that are formed by the family of rays, tangent to the caustic surface. Therefore, apart from the parabolic trajectory, SABs can follow several other caustic trajectories, mentioning couple can be bi-quadratic and natural logarithmic. For illustration purpose, we design a metasurface embedding the phase distribution of SAB (Fig. 4(c)) propagating along the natural logarithmic trajectory (*x* = ±*a*_1_ ln(*bz*)). The derived phase equation is given as:6
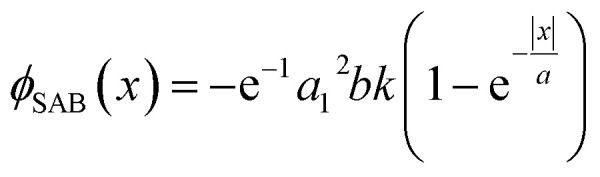
Here, *a*_1_ = 10 and *b* = 0.1. From Fig. 4(d), we can notice self-accelerating and non-diffracting natural logarithmic SAB.

**Fig. 5:**
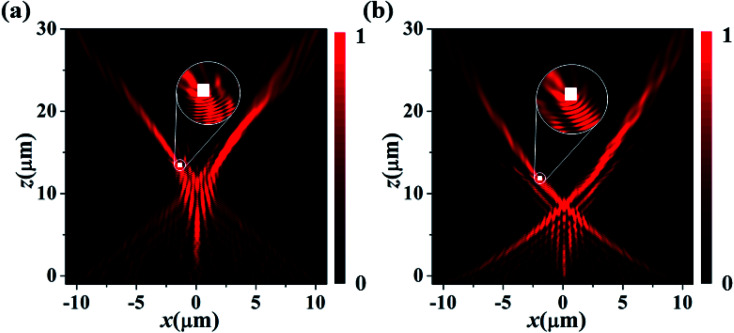
Verification of self-healing property of (a) parabolic and (b) natural logarithmic SABs. White box indicates obstruction, composed of perfect electric conductor (PEC) placed in the path of main lobes.

In the last, a riveting property of the SAB, which is the self-healing, is demonstrated. This attribute allows it to reform itself on encountering any obstruction during its propagation. To verify this property, a scatterer composed of perfect electric conductors (PEC) (shown as a white rectangular box in Fig. 5) is placed in the path of mains lobes of parabolic and natural logarithmic SABs. The size of the scatterer is kept at *P* × *P* × *P* nm^3^, where, *P* = 182 nm. From the simulated electric field distribution presented in Fig. 5(a) and (b), it can be seen that main lobes recover smoothly after passing the scatterer.

## Conclusions

In summary, we have designed and numerically simulated simple, highly efficient and polarization-insensitive all-dielectric metasurfaces capable of generating highly controllable SABs at 355 nm (UV domain). The proposed design utilizes Nb_2_O_5_ nano-rods to manipulate the phase of an impinging wave. The generated non-diffracting and self-healing SABs can propagate along any predefined propagation trajectories. Unlike conventional accelerating beam, SABs possess symmetric intensity pattern, that can manipulate multiple particles simultaneously. Furthermore, the numerical simulation confirms that these beams have a thin needle-like structure in their intensity distribution and demonstrate the autofocusing feature. We anticipate that such captivating features can have potential applications in the field of optical manipulation.

## Conflicts of interest

The authors declare no conflicts of interest.

## Supplementary Material
